# Continuous versus intermittent endotracheal cuff pressure control for the prevention of ventilator-associated respiratory infections in Vietnam: study protocol for a randomised controlled trial

**DOI:** 10.1186/s13063-018-2587-6

**Published:** 2018-04-04

**Authors:** Vu Quoc Dat, Ronald B. Geskus, Marcel Wolbers, Huynh Thi Loan, Lam Minh Yen, Nguyen Thien Binh, Le Thanh Chien, Nguyen Thi Hoang Mai, Nguyen Hoan Phu, Nguyen Phu Huong Lan, Nguyen Van Hao, Hoang Bao Long, Tran Phuong Thuy, Nguyen Van Kinh, Nguyen Vu Trung, Vu Dinh Phu, Nguyen Trung Cap, Dao Tuyet Trinh, James Campbell, Evelyne Kestelyn, Heiman F. L. Wertheim, Duncan Wyncoll, Guy Edward Thwaites, H. Rogier van Doorn, C. Louise Thwaites, Behzad Nadjm

**Affiliations:** 1 Wellcome Trust Asia Programme, Oxford University Clinical Research Unit, Hanoi, Vietnam; 2 Department of Infectious Diseases, Hanoi Medical University, Hanoi, Vietnam; 3 Nuffield Department of Clinical Medicine, Centre for Tropical Medicine and Global Health, University of Oxford, Oxford, UK; 4 Wellcome Asia Programme, Oxford University Clinical Research Unit, Ho Chi Minh City, Vietnam; 5 The Hospital for Tropical Diseases, Ho Chi Minh City, Vietnam; 6 Trung Vuong Hospital, Ho Chi Minh City, Vietnam; 7 National Hospital for Tropical Diseases, Hanoi, Vietnam; 8 Department of Medical Microbiology, Radboud University Medical Centre, Nijmegen, The Netherlands; 9 Department of Adult Critical Care, Guy’s and St Thomas’ NHS Foundation Trust, St Thomas’ Hospital, London, UK

**Keywords:** Intensive care unit, Ventilator-associated pneumonia, Intubation, Tracheal tube cuff pressure, Hospital-acquired infection

## Abstract

**Background:**

Ventilator-associated respiratory infection (VARI) comprises ventilator-associated pneumonia (VAP) and ventilator-associated tracheobronchitis (VAT). Although their diagnostic criteria vary, together these are the most common hospital-acquired infections in intensive care units (ICUs) worldwide, responsible for a large proportion of antibiotic use within ICUs. Evidence-based strategies for the prevention of VARI in resource-limited settings are lacking. Preventing the leakage of oropharyngeal secretions into the lung using continuous endotracheal cuff pressure control is a promising strategy. The aim of this study is to investigate the efficacy of automated, continuous endotracheal cuff pressure control in preventing the development of VARI and reducing antibiotic use in ICUs in Vietnam.

**Methods/design:**

This is an open-label randomised controlled multicentre trial. We will enrol 600 adult patients intubated for ≤ 24 h at the time of enrolment. Eligible patients will be stratified according to admission diagnosis (180 tetanus, 420 non-tetanus) and site and will be randomised in a 1:1 ratio to receive either (1) automated, continuous control of endotracheal cuff pressure or (2) intermittent measurement and control of endotracheal cuff pressure using a manual cuff pressure meter. The primary outcome is the occurrence of VARI, defined as either VAP or VAT during the ICU admission up to a maximum of 90 days after randomisation. Patients in both groups who are at risk for VARI will receive a standardised battery of investigations if their treating physician feels a new infection has occurred, the results of which will be used by an endpoint review committee, blinded to the allocated arm and independent of patient care, to determine the primary outcome. All enrolled patients will be followed for mortality and endotracheal tube cuff-related complications at 28 days and 90 days after randomisation. Other secondary outcomes include antibiotic use; days ventilated, in ICU and in hospital; inpatient mortality; costs of antibiotics in ICU; duration of ICU stay; and duration of hospital stay.

**Discussion:**

This study will provide high-quality evidence concerning the use of continuous endotracheal cuff pressure control as a method to reduce VARI, antibiotic use and hospitalisation costs and to shorten stay.

**Trial registration:**

ClinicalTrials.gov, NCT02966392. Registered on November 9, 2016. Protocol version: 2.0; issue date March 3, 2017.

**Electronic supplementary material:**

The online version of this article (10.1186/s13063-018-2587-6) contains supplementary material, which is available to authorized users.

## Background

Hospital-acquired infections (HAIs) are the most common adverse events affecting hospitalised patients; over 700,000 occur in the United States every year, impacting length of hospital stay, cost and mortality [1]. A point prevalence survey of HAI among nearly 20,000 patients from 23 European countries in 2010 showed an HAI prevalence of 7.1%, with the highest prevalence being in intensive care units (ICUs) (28.1%) [2]. Pneumonia and other lower respiratory tract infections were the most common types of HAI, accounting for 25.7% of all HAIs [2]. HAIs were also the second most frequent indication for antibiotic treatment, with 24% of all antibiotic use being for this indication. Respiratory infection accounted for 24.8% of HAI-related antibiotic use [2]. Data on HAIs for low- and middle-income countries (LMICs) are sparse, but they indicate that the problem is even greater in this setting with the highest burden in ICUs [3]. In Vietnam HAI prevalence in ICUs has been estimated to be as high as 30%, with up to 80% attributed to pneumonia and lower respiratory tract infection. High rates of broad-spectrum antibiotic use have resulted in very high levels of antibiotic resistance, especially among gram-negative bacteria [4], further necessitating the use of expensive, broad empirical therapy for HAI and contributing to the problem of antibiotic resistance. Most HAIs acquired on ICUs are ventilator-associated respiratory infections (VARIs) [1, 3]. A substantial reduction in this would provide direct benefit to patients and their families through shorter stay, reduced costs and potentially reduced mortality. Importantly, it would also reduce antibiotic use, a key driver of antimicrobial resistance.

Critical in the pathogenesis of lung infections in intubated patients is the movement of contaminated oropharyngeal secretions into the lungs [5, 6]. This may occur through passage internally, down the lumen of the tube, or externally around the outside of the tube, bypassing the endotracheal tube cuff. To address this, several strategies have been trialled, such as antibiotic decontamination of the oropharynx [7] (to address internal and external routes) and intermittent or continuous suctioning of secretions (subglottic suctioning, to address external routes) [8]. However, the use of antibiotic decontamination of the oropharynx, often with colistin, may cause problems in settings where infection control is limited and antibiotic resistance has reached the point that colistin is the only remaining option for many infections, or where colistin resistance among gram-negative bacteria has already emerged or been selected for. In such settings the generation and further spread of colistin-resistant gram-negative bacteria would be catastrophic, both locally and internationally. Additionally, both oropharyngeal decontamination and intermittent suctioning are labour-intensive and difficult to implement in settings where nurse-to-patient ratios are low.

Temporary falls in endotracheal cuff pressure may be a critical factor for the entry of upper airway secretions into the distal airways and lungs [6] and under-inflation of the endotracheal cuff has been associated with increased incidence of ventilator-associated pneumonia (VAP) in observational studies, with one study showing an OR of 4.3 for VAP in those patients with cuff pressure consistently < 20 mmHg [9]. Continuous pressure control (CPC) minimises the periods of low cuff pressure, preventing leakage of accumulated secretions that have pooled above the cuff, whilst also preventing high pressure that can cause tracheal injury. CPC is performed with devices that are widely used in the United States and Europe, with several devices available in Vietnam, although data supporting their use as a means of reducing VARI or VAP are relatively sparse. These devices use one of two means to maintain endotracheal cuff pressure at a given level: (1) several commercially available devices use an electric pump and electronic pressure sensor to monitor and adjust cuff pressure on a moment-to-moment basis, and (2) other more recently available devices use a pneumatic system to maintain cuff pressure by applying a set pressure through the pilot balloon and cuff inflation tubing. At the time of this writing there have been three published studies that have explored the role of CPC with conventional polyvinylchloride cuffs to reduce VAP. On one hand, a small randomised controlled trial in Spain showed that whilst CPC using a modified aquarium pump did prevent falls in cuff pressure, this was associated with a small but non-significant (from 29% to 22%, *p* = 0.44) lowering of the incidence of clinical VAP [10]. On the other hand, a quasi-randomised prospective observational study showed a reduction in VAP from 22% to 11.2% (*p* = 0.02) associated with CPC using a commercially available electronic cuff pressure controller [11]. Also, a small randomised controlled trial in a single centre in France showed a substantial and significant reduction in the occurrence of VAP during ICU stay from 26.2% to 9.8% (*p* = 0.032) associated with a pneumatic cuff pressure controller providing CPC [12]. The designs of these studies were not optimal; all had small sample sizes, one used a very rudimentary device design, only one looked at impact on antibiotic use overall and none had blinded endpoint assessment despite a clear risk of bias with unblinded endpoints using subjective criteria. Despite these limitations, CPC appears to show the most promise as a simple intervention with minimal impact on routine nursing practice to reduce VARI, though higher-quality data are required to demonstrate its impact conclusively.

To achieve a good understanding of the value of this intervention and its cost-effectiveness, patients in the present trial will be randomised to receive either automated continuous or manual control of cuff pressure three times per day (current standard practice). This trial will produce high-quality data to inform the future care of ventilated patients in Vietnam and throughout the world.

## Methods/design

### Aims of the study

The aim of this trial is to determine if CPC can reduce the incidence of VARI in adults. Secondary aims include establishing whether CPC leads to an increase in proportion of ICU days not on antibiotics or a reduction in antibiotic costs, a reduction in the frequency of HAI as a whole during ICU stay, length of ventilation, length or cost of ICU and hospital stays, as well as any local complications of the endotracheal cuff and mortality at 28 and 90 days post-randomisation and at discharge from ICU and hospital.

### Study design

The proposed study is an open-label, randomised controlled trial comparing tracheal cuff CPC via an automated electronic device with intermittent manual pressure control for the prevention of VARI in ICU. For the purpose of this study a stand-alone CPC device (reference 701; TRACOE medical GmbH, Nieder-Olm, Germany) will be used for the intervention.

### Study population

Patients are being recruited over an 18-month period from the ICUs in the Hospital for Tropical Diseases (HTD) and Trung Vuong Emergency Hospital (TVH) in Ho Chi Minh City and the National Hospital of Tropical Diseases (NHTD) in Hanoi. HTD and NHTD are referral centres for the management of patients with infectious diseases. Both receive patients with infectious diseases directly from their local populations as well as patients transferred from other hospitals in southern and northern Vietnam. TVH is a provincial hospital serving the general medical needs of the local population in Ho Chi Minh City.

Patients are considered eligible for inclusion in the study when they are at least 18 years of age, have been intubated for ≤ 24 h at the time of randomisation (either oral or tracheostomal) and for active treatment (i.e., physician caring for patient would prescribe an antibiotic if the patient developed an infection). Exclusion criteria are previous enrolment in the study; having been previously intubated within the last 14 days; and known tracheal stenosis, tracheomalacia or stridor that is suspected secondary to physical tracheal injury. Patients are enrolled only following written informed consent provided by themselves or their legal representatives.

Patients are stratified by clinical diagnosis as tetanus or non-tetanus, because patients with tetanus represent a distinct subgroup; usually they are without significant premorbid disease or systemic inflammatory response, undergo primary tracheostomy and have a prolonged duration of intubation and ICU stay. Whilst rare in high-income settings, in many countries patients with tetanus represent a significant burden of disease with high rates of HAI and high consumption of ICU resources [13, 14]. Thus, a protocol that attempts to address the problem of HAI in this setting also needs to specifically address tetanus.

### Study endpoints

The primary endpoint is the occurrence of VARI which is defined as VAP or ventilator-associated tracheobronchitis (VAT) during ICU stay up to a maximum of 90 days post-randomisation as evaluated by an independent endpoint committee. For both VAP and VAT, it is a core requirement for the patient to have been intubated for at least 48 h and for the tube to have been in place within the 48 h before the infection occurred. An additional core criterion for all cases is that a decision has been made to start new antibiotics or change the antibiotic regimen to treat a new infection. In addition to these core criteria, the endpoint diagnosis of VAT further requires new onset of purulent respiratory secretions or change in character of sputum or increase in volume of sputum plus either (1) temperature > 38 °C or < 36 °C or (2) white blood cell count < 4.0 × 10^9^/L or ≥ 12 × 10^9^/L with no other recognised cause. The endpoint diagnosis of VAP is met when the core criteria are met and there are new or progressive changes visualised by chest radiography plus two of following three criteria: (1) temperature > 38 °C or < 36 °C, (2) white blood cell count < 4.0 × 10^9^/L or ≥ 12 × 10^9^/L with no other recognised cause, and (3) a new onset of purulent respiratory secretions or change in character of sputum or increase in volume of sputum.

Secondary endpoints include microbiologically confirmed VARI (defined as VARI plus bacterial growth of ≥ 10^5^ colony-forming units/ml [endotracheal aspirate] or equivalent semi-quantitative growth), clinical and microbiologically confirmed VAP, proportion of intubated days receiving antibiotics, incidence of other HAI (as defined by the European Centre for Disease Prevention and Control [ECDC] criteria [15]) whilst intubated, total number of days ventilated/in ICU, cost of ICU/hospital stay, cost of antibiotics during ICU/hospital stay and mortality at 28 and 90 days after randomisation as well as at ICU and hospital discharge. Safety data, including in-hospital re-intubation, tracheomalacia, tracheal stenosis and other local complications of endotracheal placement, will be collected up to 90 days post-enrolment or until hospital discharge, whichever is later. The time periods over which endpoints or censoring events will be elicited are shown in Table 1.

**Table 1:** Follow-up periods for all study endpoints

Endpoint	Beginning of follow-up	End of follow-up/censoring event (soonest event applies, except where specified)
Primary		
VARI	Randomisation	ICU discharge/death/transfer or 90 days
Secondary		
Microbiologically confirmed VARI	Randomisation	ICU discharge/death/transfer or 90 days
Clinical and microbiologically confirmed VAP	Randomisation	ICU discharge/death/transfer or 90 days
Intubated days not receiving antibiotics	Randomisation	ICU discharge/death/transfer or 90 days
Incidence of HAI	Randomisation	Extubation/death/transfer/discharge from ICU or 90 days
Days ventilated/in ICU	Randomisation	ICU discharge, death, transfer
Cost of ICU stay	ICU admission	ICU discharge/death/transfer
Cost of antibiotics in ICU stay	ICU admission	ICU discharge/death/transfer
Cost of hospital stay	Hospital admission	Hospital discharge
28-Day mortality	Randomisation	28 Days after randomisation
90-Day mortality	Randomisation	90 Days after randomisation
ICU mortality	Randomisation	Discharge from ICU or death/palliative discharge from ICU
Hospital mortality	Randomisation	Discharge from hospital or death/palliative discharge from hospital

*Abbreviations: VARI* Ventilator-associated respiratory infection, *ICU* Intensive care unit, *VAP* Ventilator-associated pneumonia, *HAI* Hospital-acquired infection

### Sample size

Based on local data and data derived from a large point prevalence study in Vietnam [4], the estimated prevalence of VARI (the primary outcome) in non-tetanus and tetanus ventilated patients is 20% and 30%, respectively. We expect an effect size of a 50% reduction in VARI using CPC, but we consider that a 40% reduction would be the minimum we would wish to detect [11, 12]. This effect size is expected to be the same in both tetanus and non-tetanus patients. To preserve the generalisability of the study for settings where tetanus is less common whilst still demonstrating the utility of the intervention in this patient group, we will stratify randomisation to ensure that 30% of our recruited patients will have a diagnosis of tetanus. Thus we might expect to reduce VARI rates from 20% to 12% in non-tetanus patients and from 30% to 18% in tetanus patients. In a study population with 30% tetanus patients, this corresponds to an absolute risk reduction from 23% to 13.8%. To detect this reduction with 80% power at the two-sided 5% significance level, 278 patients are required in each arm. Allowing for 8% loss to follow-up or early extubation (before 48 h), we aim to recruit 600 patients in total (420 non-tetanus, 180 tetanus). To our knowledge, this will make it the largest single trial addressing the prevention of VARI through CPC.

### Randomisation procedure

Randomisation will be stratified 1:1 by site and whether the patient has a clinical diagnosis of tetanus at the time of randomisation. A stratified, computer-generated randomisation list will be created using block randomisation with variable block length and incorporated into secure internet-accessible software that implements the randomisation. Once an eligible patient has consented, the initials and date of birth of the patient will be entered into the software by study staff. On the basis of the randomisation list, the software will produce the treatment allocation, which will be displayed and recorded in the study database. All entries and outputs of the software will be auditable.

### Intervention

Depending on the results of randomisation, patients will receive either manual, intermittent endotracheal cuff pressure, which is checked and adjusted 8-hourly (standard care), or automatic, continuous endotracheal CPC (intervention group). Cuff pressure will be recorded in every ventilated patient every 8 h. Target pressure in both groups will be 25 cmH_2_O as a default. Changes in this target will be recorded, and the clinical reason for the change will be noted.

### Study assessment

On a daily basis, the attending physicians will assess whether there is a new infection in a patient who has been intubated for ≥ 48 h and in whom the tube is still in place or has been removed within the previous 48 h. If the answer is ‘yes’, a standard battery of tests, including complete blood count, procalcitonin, arterial blood gas, blood culture, sputum/endotracheal aspirate microscopy and culture, urine culture and chest x-ray, will be performed regardless of the suspected site of infection. Test results and clinical details from up to 5 days previous, the day of the HAI evaluation and the subsequent 2 days will be collected, including maximum/minimum temperature, changes in ventilation parameters (fraction of inspired oxygen, positive end-expiratory pressure), changes in sputum colour or volume, and new inotrope or vasopressor requirements. The treating clinicians will provide their own diagnosis of the aetiology of the infection according to ECDC criteria and will prescribe antibiotic and other therapies as per routine care. However, for the primary endpoint, an endpoint review committee, blinded to the treatment allocation and independent of clinical involvement with the patient, will review the complete case report form (CRF) and radiology of patients completing the study at the end of each month to determine whether they met the primary or appropriate secondary endpoint criteria.

**Fig. 1:**
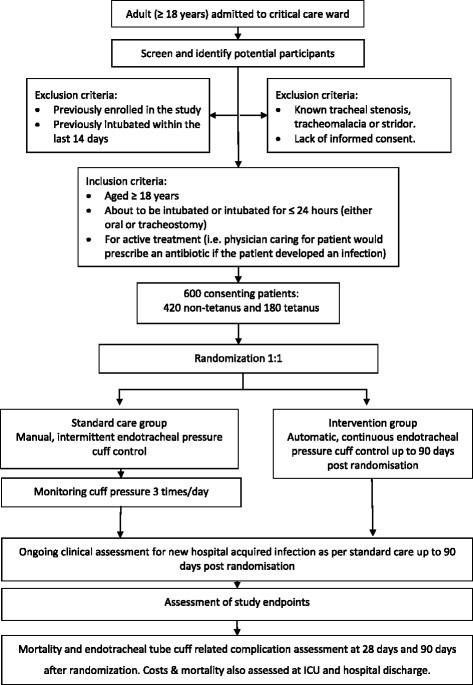
Flowchart of study procedures

**Fig. 2:**
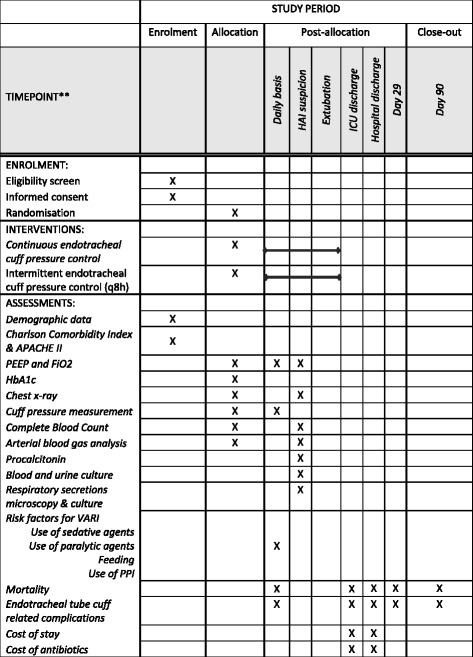
Schedule of enrolment, interventions and assessments. *HAI* Hospital-acquired infection, *ICU* Intensive care unit, *APACHE* Acute Physiology and Chronic Health Evaluation, *PEEP* Positive end-expiratory pressure, *FiO*_*2*_ Fraction of inspired oxygen, *HbA1c* Glycated haemoglobin, *VARI* Ventilator-associated respiratory infection, *PPI* Proton pump inhibitor

Enrolled patients will have cuff pressure controlled in accordance with the allocated study arm for the entire duration of their intubated time on ICU. Follow-up for the primary outcome will be until transfer to another facility, death, discharge from ICU or 90 days following randomisation, whichever is soonest. Patients who require re-intubation during the same stay in ICU will continue in the study, using the same cuff pressure control measures originally allocated to them. Patients discharged from ICU but requiring return to ICU and re-intubation will not be re-enrolled and will be managed in accordance with standard care. At the end of the hospital stay the costs of the stay as billed will be recorded (including bed, ventilator and other supportive therapy costs, drugs including antibiotic costs, but not including labour as per Vietnamese Ministry of Health guidance). Additionally, as part of secondary endpoint assessment, study staff will telephone patients or their relatives at 28 and 90 days after randomisation if they are no longer inpatients in order to assess mortality and complications related to intubation. A flowchart of study procedures is shown in Fig. 1; a schedule of enrolment, interventions and assessments is provided in Fig. 2.

### Data management

Relevant data will be recorded onto a CRF and checked for accuracy. After the follow-up is finished the CRFs will be de-identified by removing patient identifiers and telephone numbers. Selected staff will be trained in how to enter all clinical data from the CRFs and from laboratory source documents into a computerised data entry system using double data entry. Data will be retained for ≥ 15 years on completion of the study (last patient followed for 90 days). Original paper documents will be maintained for ≥ 5 years; thereafter digital copies will be retained. All stored records will be kept secure and confidential.

All personal information reviewed as part of this study will remain confidential. Patient names or identifiers will not appear in any database. Any scientific publications or reports will not include information that can identify any patient. Oxford University Clinical Research Unit (OUCRU) is committed to open access to study data and is working with its partners to facilitate mechanisms to enable this.

### Statistical analysis

The details regarding all planned analyses will be elaborated in an analysis plan prior to analysis. Reporting of the trial results will clearly indicate which analyses were part of this plan and which were not. The effect of the intervention on the primary outcome will be quantified in an intention-to-treat analysis that allocates all patients according to the randomised treatment arm. The primary endpoint will additionally be analysed in a per-protocol analysis which will exclude patients not fulfilling the eligibility criteria and patients intubated for < 48 h.

The primary endpoint will be analysed using a logistic regression model with the randomised arm as the main covariate and adjustment for tetanus status. Patients who are intubated for < 48 h will be regarded as not having reached the primary endpoint. Potential heterogeneity of the intervention effect will be assessed on the basis of appropriate interaction (likelihood ratio) tests and the predefined subgroups, including (1) patients with and without tetanus, (2) patients intubated for ≤ 2 and > 2 h before randomisation, (3) patients with and without tracheostomy and (4) hospital site.

Binary secondary endpoints (clinical and microbiologically confirmed VAP) will be analysed in the same way as the primary endpoint. Both the distribution of duration of ventilation (i.e., time to extubation) and the time of ICU stay will be estimated. Death will be considered as a competing event; hence we will estimate the cause-specific cumulative incidence. Cause-specific cumulative incidence functions will be compared between the arms using a Fine-Gray regression model with the treatment arm as the main covariate and adjustment for tetanus status. Mortality will be visualised in each arm (overall and by tetanus status) using Kaplan-Meier curves and modelled using Cox regression. The proportion of intubated days free of antibiotics will be analysed using a Poisson regression model with the number of intubated days without antibiotics as the outcome, the randomised arm as the main covariate, and the (log-transformed) total number of intubated days as an offset. Quasi-likelihood will be used to account for potential over-dispersion.

The frequency of adverse events will be summarised (in terms of both the total number of events and the number of patients with at least one event). The proportion of patients with at least one adverse event (overall and for each specific event separately) will be summarised and (informally) compared between the two treatment groups using Fisher’s exact test.

### Reporting of adverse events and monitoring

Because adverse events are relatively common in critically ill patients intubated in the ICU, safety reporting will focus on events of potential relevance to cuff pressure control. The events being reported to the ethics committees are (1) all unexpected serious adverse events, (2) all serious adverse events judged to be related or possibly related to cuff pressure and (3) all deaths or palliative discharges (discharge to home of a patient where ongoing care was considered futile to permit the patient to die at home, a commonly preferred alternative to dying in hospital in Vietnam). Reporting to the ethics committees will be done in accordance with good clinical practice.

An independent data and safety monitoring board (DSMB) has been established, consisting of expert Vietnamese and international researchers and doctors with the necessary clinical, research and statistical knowledge. The DSMB has reviewed the protocol, and a DSMB charter outlines its responsibilities and how it operates. All DSMB reports will be sent to the responsible ethics committees, including the site ethics committees and the Oxford Tropical Research Ethics Committee, for consideration. Recruitment will continue at active sites during the DSMB review period. No interim analyses are planned for the primary endpoint; however, safety analyses will be conducted after 30 patients have been recruited and subsequently at points decided by the DSMB.

The study will be monitored by a team at OUCRU, independent of study design and management. Central monitoring will take place regularly. On-site monitoring occurred after the recruitment of 25 patients in total, with a second monitoring visit planned after recruitment of 200 patients, and further monitoring will be scheduled depending on the results of early monitoring visits. The SPIRIT (Standard Protocol Items: Recommendations for Interventional Trials) for this research is available in Additional file 1.

## Discussion

To our knowledge, this is the first study with an adequate sample size and robustly designed endpoints to evaluate the effectiveness of endotracheal cuff CPC in preventing VARI. It also concurrently measures the impact of the intervention on important patient-centred clinical outcomes and important outcomes concerning antibiotic use. An ideal intervention should, in addition to an impact on the primary outcome (VARI episodes), show resultant benefits in reducing mortality, duration of mechanical ventilation and ICU stay in association with reducing antibiotic use, as recently recommended for antibiotic stewardship programmes [16]. The intervention will likely be considered of questionable benefit should it result only in a reduction in VARI episodes without corresponding reductions in at least some of these patient-centred or antibiotic stewardship outcomes.

Leakage of secretions through the endotracheal tube cuff is commonplace in clinical practice with intermittent cuff pressure monitoring [17]. Approaches to prevent aspiration of contaminated oropharyngeal secretions by maintaining endotracheal cuff pressure have been studied in an effort to reduce VAP rate and its associated mortality. However, previous studies (small and non-randomised controlled trials) have not established a sufficient evidence base on which to recommend CPC routinely [18, 19]. This study provides an opportunity to evaluate the impact of CPC in preventing VARI and in reducing antibiotic use as an aspect of antimicrobial stewardship in a resource-constrained setting with established high levels of antibiotic use and resistance.

Pathogen colonisation of the trachea and endotracheal tube may progress to VAT, which can be a transitional stage before development of VAP. Both VAT and VAP share common clinical manifestations of fever, purulent respiratory secretions, and leucocytosis with the distinguishing presence of pulmonary infiltrates on chest x-rays in VAP. In a prospective study in 10 countries with 114 ICUs, incidence of VAT was similar to VAP (11% vs 12%) [20]. However, VAT is often a predisposing factor for development of VAP, and appropriate antibiotic treatment for VAT can reduce the progression to VAP [20, 21]. In a survey of perceptions of international physicians, nearly 80% of respondents diagnosed VAT on the basis of both clinical and microbiological criteria, and the remaining respondents used clinical criteria alone or as a diagnosis of exclusion. Half of the respondents agreed to antibiotic use in patients with VAT [22]. In Vietnam it is common practice to treat VAT, and in view of these considerations, we constructed our trial around an endpoint that encompassed both VAT and VAP, in contrast to other studies [10–12]. Although recognition of VAT and intervention at this stage are expected to impact the mortality, length of stay and treatment cost which would be attributed to VAP, it is clear that there are important differences between surveillance definitions and actual clinical definitions on which treatment is based.

In an open-label study where the treating doctor is not blinded to the intervention, there is a risk of clinical ascertainment bias of the study endpoint [23]. Whilst death or microbiological results are hard endpoints which are unlikely to be biased by a lack of blinding, VARI diagnostic categories and antibiotic use are more subjective and vulnerable to misclassification bias in non-blinded trials [24]. For this reason an independent endpoint committee is recommended for multicentre clinical trials to judge complex, multi-aspect definitions and outcomes [25, 26]. In our study treating doctors know the allocated arm of the patient and perform assessments for HAI as part of routine care; therefore the use of a blinded endpoint committee will reduce bias in the primary endpoint evaluation. The study may also offer an opportunity to understand inconsistencies in the assessment of VARI diagnoses between treating doctors and the endpoint committee, and within the endpoint committee, as well as how this impacts the significance of results.

A future consideration would be to evaluate the impact of a successful intervention at the ICU level, on the speed at which resistance develops in the unit, with the hope that a substantial reduction in the use of antibiotics would be reflected in slower development of resistance in the ICU as a whole. This would require a cluster-randomised or stepped-wedge design, and such an approach should be considered for any potential rollout of a successful intervention. Given the crisis in antibiotic resistance, this would provide valuable additional information not attainable through an individual patient randomised trial design to complement the results of this study.

### Trial status

The trial is currently recruiting. Important protocol amendments will be communicated to relevant bodies (investigators, institutional review boards, DSMB, journals) by Dr. B. Nadjm, trial principal investigator, as soon as changes are made.

### Trial *s*ponsor

Oxford University - Centre for Tropical Medicine (sponsor’s reference R45398/RE001). Contact: Evelyne Kestelyn, 764 Vo Van Kiet Street, Ward 1, District 5, Ho Chi Minh City, Vietnam. Telephone: + 84 8 39237954. Email: info@oucru.org. The sponsor has specialised insurance to cover any potential harm caused as a result of participation in the study.

## Additional file

Additional file 1: Standard Protocol Items: Recommendations for Interventional Trials (SPIRIT) 2013 checklist: recommended items to address in a clinical trial protocol and related documents. (DOCX 51 kb)
